# A Practical and Historical Treatise on Consumptive Diseases, Deduced from Original Observations, and Collected from Authors of All Ages

**Published:** 1817-03

**Authors:** 


*A Practical and Historical Treatise on Consumptive Diseases, deduced from original Observations, and collected from Authors of all Ages.* By Thomas Young, M. D. F. R. and L. S. Fellow of the Royal College of Physicians, and Physician to St. George's Hospital. 8vo. pp. 469. London, 1815.

By all who, in these islands, cultivate Medicine as a branch of philosophy, or practice it as a science, the name of Dr. Young must have been long familiarly known, and his talents duly appreciated. If the views, in which we are accustomed to regard the constitution of the medical character be correct, there are few men to whom the title of the enlightened physician, a title comprehending such rare combinations of native talent and acquired knowledge, can be justly applied; and, among those few, we hesitate not to rank Dr. Young. His "Medical Literature," published about three years since, though like all other productions of the human mind, in some respects faulty, constitutes most honourable evidence of its author's ability and erudition; nor is it calculated to disgrace the shelf whereon repose the costly and elaborate publications of Haller and Plocquet.

The present Treatise, rather comprehending all that has been written by others on the subject of consumptive diseases, than disclosing original views of their history, pathology, or treatment, will certainly not detract from the reputation of Dr. Young. It consists of a Preface; and of two books, respectively entitled *Practical Observations* and *Medical History*. The latter, we regret to say, will rarely admit of analysis.

*Preface.* The work, we are here informed, is destined to comprehend every important fact which Dr. Young has himself observed, or been able to find recorded, "with respect to the nature and cure of the diseases belonging to a single genus." The information, afforded by his medical literature will facilitate the execution of a similar project in every other disease; and the interest of science, it must be allowed, are more effectually promoted by such concentration of labour, than by its expenditure on a wider and more diversified field. We are sorry, however, that Dr. Young has relinquished the large and general work on medicine which he once had in contemplation. A sound and comprehensive system of practical physic is, like one of anatomy, yet a desideratum in the literature of our country; and we know no one better qualified than Dr. Young to fill up the void with honour to himself and advantage to the science.

BOOK I, entitled *Practical Observations,* consists of seven Chapters.

CHAPTER 1. *Symptoms of Hectic Fever.* Although sometimes idiopathic, this fever may be, for the most part, regarded as dependent on some other local or constitutional affection: in either case, the fever will form a better criterion whereby to appreciate the degree of constitutional derangement, and chance of recovery, than any other assemblage of symptoms. Upon this principle, Dr. Young in his nosological arrangement, assumes *Hectica* as the title of a genus, which comprehends three species: *Hectica debilium,* simple hectic, without emaciation; *Hectica tabes,* decline or wasting without pulmonary affection; and *Hectica phthisis,* or consumption with cough. Frequent weak pulse, flushings in the face, hands, or feet, and profuse night sweats, constitute the essential character of this fever. The fever itself is remittent, never completely intermittent; the pulse sometimes hard and wiry though small, especially where tendency to inflammation exists, and the remissions are less distinctly marked: the elevation of thermometrical temperature is commonly considerable. The tongue is rarely so much furred as in other febrile affections, its margins being generally of a bright red, and its papillæ prominent; but, if the biliary system be deranged, it usually exhibits a white coat. The digestive energies continue unimpaired, and the appetite good, to the last. The principal febrile exacerbation commonly takes place towards evening (in some rare instances in a morning) and a slighter paroxysm about noon; but a full meal, taken at any hour, seems to induce its recurrence. The exacerbation is ushered in by a sense of chilliness, although no actual reduction of temperature has yet been ascertained; marked by burning heat in the palms of the hands, and circumscribed redness of the cheeks; and succeeded towards the close of night by copious sweats, general or partial; which disappear on the supervention of diarrhœa, or alternate with it: though the cuticular discharge does not invariably recur when the intestinal is suppressed. The urine, in general very frequently deposits a reddish sediment of uric acid, or a light branny one, after the sweat, and not during the hot fit; when the secretion is commonly pale and limpid; but this phænomenon is very irregular as to the time of its occurrence, sometimes altogether unobserved. Defective nutrition, as the disease advances, is signalized by the falling off of the hair, and curvature of the nails. In many cases, there are irregular pains in the limbs, and ulceration of the integuments from pressure; and occasionally severe head-ache, progressively increasing in violence throughout the whole course of the disease. Of the remote causes of hectic, little is known. The exciting cause is commonly a local affection, and that, for the most part, incurable. Sometimes, the symptoms seem to be induced by a particular change in the secretions of morbid parts, as the exposure of a purulent collection to the air; but hectic may occur independently of abscess, and is seldom excited by the unhealthy suppuration of cancer. When dependent on local affection, its symptoms will be generally proportioned to the severity of the latter and often promptly disappear on removal of the diseased part.

Chapter 2. *Symptoms of Decline.* The first species of *Hectica*, or *H. debilium*, nearly corresponding to the slow fever of the ancients, is of rare occurrence, and requires no practical discussion, except as a stage, or at most a variety, of one of the other species. The varieties of the second species, *H. tabes*, are (although, if the evidence of anatomy only were consulted, they would become numerous as the diversities of local affection) four: A. *Simple decline*, independent of organic disease, and subdivided into a, *juvenile decline*, occurring about the age of puberty, with pains in the limbs and swellings of the joints; b, *adult decline*, in middle age, with cephalic pains, aphthæ, nausea, diarrhœa; and c, *senile decline*, the marasmus of Hoffman, with dry mouth, hoarseness, parched skin, hard pulse and sleeplessness: — B, *Intesti-*

*nal decline*, with languid action of the bowels, resembling the infantile fever of Sydenham, and the marasmus of Hamilton, accompanied by great irritability, restlessness, anorexia, or sometimes craving appetite, and general debility: C, complicated with tumour, and especially enlarged glands. The affections, composing this variety, and the following, belong properly to the genera under which the respective local diseases would be classed, the attendant fever being so far symptomatic as always to require the application of means immediately directed to remove the primitive malady: while in consumption a plan of treatment, rather general than local, is required. Yet some of these affections are so closely allied to the latter, that, in their symptoms and treatment, they serve well to illustrate it. Of these, the principal is *tabes mesenterica*: its symptoms are head-ache, languor, anorexia; acute pain in the back and loins; fullness, then pain and tenderness of the abdomen; chalkiness and want of consistence of the fæces, from obstruction of the chyle and deficiency of the biliary secretion, sometimes mixed with blood and mucus, and their discharge attended by the irritation and tenesmus of dysentery; frequently dropsical effusion, particularly ascites; appetite, in the more advanced stages, good, often ravenous. It it sometimes incorrectly attributed to the presence of intestinal worms. Though dangerous, it is much less intractable than genuine phthisis: D, *The hectic of suppuration* may be discriminated from others, as rarely occurring until the pus of the abscess has been exposed to the external air; whereby it is believed to acquire an acrimonious quality, inducing by irritation, the symptoms of hectic. In this variety are included the hectic affections arising from suppuration of the several important organs of the animal economy.

Chapter 3. *Symptoms of Consumption.* With these, our readers, both from description and personal observation, must be so familiarly acquainted that we shall not pause to review the melancholy catalogue. There are, however, some means of ascertaining the approach or progress of the malady which many among them may have never heard of, or forgotten; and which it may, therefore, be proper cursorily to notice. Of these diagnostic means, three have for their object the determination of the capacity or state of the chest; a fourth, the establishment of the long-sought-for criterion between pus and mucus. The first was originally proposed by Mr. Abernethy, and consists in causing the patient, after a *full* inspiration, to breathe, through a bent tube, into an inverted jar filled with water. The quantity of fluid displaced, which may be known by marking the side of the jar, and subsequently filling it with water to that point, will indicate the volume of air expired; and, if there be much less than six quarts in the adult, some part of the organs concerned in respiration is certainly diseased. Upon the second, or percussion of the chest, Dr. Young seems to place but little value; and we may add, that our own evidence respecting its employment in tubercular phthisis is decidedly unfavourable: in the apostematous form of the disease alone, and then only when the collection of pus was very considerable, have we been able to obtain from the sound of the chest any clear and satisfactory results. In the third mode of examination, it must be observed whether, on a deep inspiration of the patient, the sternum rise naturally or remain nearly fixed. The lungs, in the latter case, must have lost their wonted expansibility. The last experiment, which has the matter of expectoration for its subject, is extolled by Dr. Young as "a very simple and certain optical criterion." It depends on the presence of globules, quite unconnected with admixture of blood, in purulent secretion. If a little of the substance under examination be put between two pieces of plate glass, and the observer look through it, held near his eye, at a distant candle, he will see, even by day-light, "a bright circular corona of colours, of which the candle is the centre; a red area, surrounded by a circle of green, and this again by another of red; the colours being so much the brighter, as the globules are more numerous and more equable." Should the substance be simple mucus, no "rings of colours" will be visible: yet there may exist, even in mucus, a sufficient mixture of heterogeneous particles to cause the appearance of a reddish area only about the candle." Mucus, although commonly floating in water, will sink to the bottom, when destitute of air. In some cases, attended by every other symptom of consumption, the fever has been absent.

CHAPTER 4. *Morbid Appearances in Consumption.* Sometimes, in persons dying of confirmed phthisis with purulent expectoration and diarhœa, "the lungs have exhibited scarcely any appearance of diseased structure," the expectorated pus having been derived from a morbid secretion of the bronchial membrane. In such cases, Dr. Young considers it "probable that the capacity of the chest would be less diminished than in genuine tubercular consumption;" but that their correct discrimination would be alike impracticable during the life of a patient and destitute of utility in the treatment. We are far, however, from regarding the latter of these opinions as strictly orthodox. The morbid appearances, more commonly observed upon anatomical inspection of phthisical subjects, are, the indurated bodies, called tubercles, occupying the cellular structure of the *lungs*, particularly at their superior and posterior parts; at first minute, whitish, opaque (in some rare instances transparent, cartilage-like, with black spots in their substance) gradually enlarging and forming small vomicæ, which contain a curdy half-formed pus, and sometimes leave, on its evacuation, an open cavity, sometimes collapse; or more frequently softening in the centre when they have acquired the volume of a large pea, and bursting by one or more small orifices, into the neighbouring bronchia: The bronchial glands in a state of suppuration: Simple vomicæ or abscesses, independent of previous formation of tubercles, and destitute of the living membrane which characterizes the tubercular vomica; and principally found in cases of phthisis consequent on hæmoptysis and pneumonia: Conversion of the lungs into a liver-like or even horny substance, or into a mass as black as charcoal and of the consistence of leather, displaying black ulcers, with intervening portions of sound lung. Calculous concretions, especially in the vicinity of the bronchial glands, consisting of phosphate of lime and animal matter, and hence not simply composed of particles of foreign matter which have been accumulated in respiration: Whitish tumours, of different sizes, in the lungs of cancerous subjects, resembling brain, pervaded by numerous blood-vessels, and containing, when soft, a cream-like fluid: Reduction of the lungs to the consistence of mud or clay; gangrene, scirrhus, or ossification of these organs. Thickening of the laryngeal membrane with traces of inflammation, or ulceration usually complicated with deposition of ossific matter, especially where hoarseness has formed a strongly marked symptom.* In the *intestines*, particularly in the

* M. Bayle, a respectable foreign writer on pulmonary phthisis, found the larynx diseased in one sixth of the cases which he examined; and intestinal ulceration in sixty-seven out of a hundred.

iliac portion, when the diarrhœa has been severe, inflammation and spots of ulceration, attributed, but erroneously, by some to a tubercular degeneration of the intestinal mucous glands, analogous to the changes of the cervical and mesenteric absorbent glands, which are frequently observable in the same constitution. The inflammation of the internal membrane is at first superficial, and unaccompanied with thickening, until the ulcer has formed; the margins of which become subsequently thickened: Sometimes there is thickening without ulceration. Lastly, in the *heart*, thinness and diminution of volume; and, frequently, enlargement of the foramina thebesii.

In the perusal of this chapter, to which no one is better qualified than Dr. Young, to give an interest and development commensurate with the progress of morbid anatomy, we have experienced no common disappointment. Instead of recording indiscriminately every variety of morbid appearance which has ever been observed on dissection of phthisical subjects, Dr. Young would, we imagined, have attempted to point out the peculiar lesions of structure, upon which the more strongly-marked species of pulmonary consumption are essentially dependent; and the general train of external phænomena by which they may be respectively distinguished during life. That such differences do absolutely exist, and require plans of treatment corresponding to their peculiarity of origin and character, no one, experienced, like Dr. Young, in the observation of pulmonary diseases, would, we really thought, have been found to deny. Our limits will not, at present, allow us to state a general outline of the plan upon which, we conceive, a nososological arrangement of consumptive diseases might be most correctly instituted; sanctioned alike by well defined peculiarities of external sign and internal morbid alteration. To this subject we shall probably revert at some future period.

( To be continued. )

